# The Immune Response to Melanoma Is Limited by Thymic Selection of Self-Antigens

**DOI:** 10.1371/journal.pone.0035005

**Published:** 2012-04-10

**Authors:** Ulrike Träger, Sophie Sierro, Gordana Djordjevic, Basma Bouzo, Shivani Khandwala, Antonella Meloni, Monika Mortensen, Anna Katharina Simon

**Affiliations:** 1 MRC Human Immunology Unit, Weatherall Institute of Molecular Medicine, Nuffield Department of Medicine, University of Oxford, Oxford, United Kingdom; 2 NIHR Biomedical Centre, Oxford, United Kingdom; 3 Ludwig Institute for Cancer Research, Epalinges, Switzerland; 4 Pediatric Clinic II, Ospedale Microcitemico and Department of Biomedical and Biotechnological Science, University of Cagliari, Cagliari, Italy; 5 Apoptosis Department and Center for Genotoxic Stress Research, Institute of Cancer Biology, Danish Cancer Society, Copenhagen, Denmark; Weizmann Institute of Science, Israel

## Abstract

The expression of melanoma-associated antigens (MAA) being limited to normal melanocytes and melanomas, MAAs are ideal targets for immunotherapy and melanoma vaccines. As MAAs are derived from self, immune responses to these may be limited by thymic tolerance. The extent to which self-tolerance prevents efficient immune responses to MAAs remains unknown. The autoimmune regulator (AIRE) controls the expression of tissue-specific self-antigens in thymic epithelial cells (TECs). The level of antigens expressed in the TECs determines the fate of auto-reactive thymocytes. Deficiency in AIRE leads in both humans (APECED patients) and mice to enlarged autoreactive immune repertoires. Here we show increased IgG levels to melanoma cells in APECED patients correlating with autoimmune skin features. Similarly, the enlarged T cell repertoire in AIRE^−/−^ mice enables them to mount anti-MAA and anti-melanoma responses as shown by increased anti-melanoma antibodies, and enhanced CD4^+^ and MAA-specific CD8^+^ T cell responses after melanoma challenge. We show that thymic expression of gp100 is under the control of AIRE, leading to increased gp100-specific CD8^+^ T cell frequencies in AIRE^−/−^ mice. TRP-2 (tyrosinase-related protein), on the other hand, is absent from TECs and consequently TRP-2 specific CD8^+^ T cells were found in both AIRE^−/−^ and AIRE^+/+^ mice. This study emphasizes the importance of investigating thymic expression of self-antigens prior to their inclusion in vaccination and immunotherapy strategies.

## Introduction

Melanomas account for only 4% of dermatological cancers, but are responsible for 80% of deaths resulting from skin cancer [1]. Moreover, the 5-year survival rate from metastatic melanoma is only 14% and no effective therapy is yet available against melanoma. A better understanding of melanoma immunosurveillance is therefore essential to enable the design of better, targeted melanoma therapies.

The choice of target antigens is key to the success of tumour vaccination or tumour immunotherapy. Melanoma candidate antigens include (A) mutated or aberrantly expressed molecules (e.g. CDK4, MUM-1, beta-catenin) (B) cancer/testis antigens (e.g. MAGE, BAGE and GAGE) and (C) melanoma- associated antigens (MAA) [2]. MAAs are self-antigens normally expressed during the differentiation of melanocytes and play a role in different enzymatic steps of melanogenesis. However, in transformed melanocytes (melanoma cells), MAAs are often overexpressed. The main MAAs are tyrosinase, an enzyme that catalyses the production of melanin from tyrosine by oxidation, the tyrosinase-related proteins (TRP-1) and 2 (TRP-2), the glycoprotein (gp)100 (silver-gene) and MelanA/MART. It is thought that the specialized cell biology of melanin synthesis may favour the loading of MAA peptides into the antigen presentation pathway [3]. 50% of melanoma patients have tumour-infiltrating lymphocytes (TILs) recognising tyrosinase and Melan A, indicating that these antigens are important in the natural melanoma immunosurveillance [2]. Moreover, MAAs are well characterized in mice and humans, allowing the development of tetramers to detect antigen-specific immune responses.

However, as MAAs are self-antigens, it is known that the immune system establishes immunological tolerance to them either in the thymus or in the periphery. Thymic tolerance, is achieved by the promiscuous expression of tissue-specific self-antigens by medullary TECs (mTECs) promoting self-tolerance and is controlled by the autoimmune regulator AIRE, a transcriptional regulator of several thousands of genes in mTECs [4]. However, AIRE's exact mechanisms of action are only just being elucidated. Defects in AIRE lead to multiple autoimmune disorders in mice and patients with APECED (autoimmune polyendocrinopathy-candidiasis-ectodermal dystrophy) [5]. APECED patients suffer from a mild immune deficiency leading to persistent mucosal and cutaneous infections with candida, more importantly autoimmune dysfunction is observed in many organs resulting in hypothyroidism, hypogonadism (infertility), vitiligo and alopecia.

Thymic selection is leaky and the ensuing autoimmune repertoire is kept under control by peripheral tolerance mechanisms. Passive peripheral tolerance is induced in T cells by presenting antigens under tolerogenic conditions. It has been suggested that AIRE may also play a role in peripheral expression of tissue-specific antigens [6]. Regulatory T cells (Tregs) represent another (active) mechanism of peripheral tolerance. It is also still controversial whether their positive selection is under the control of AIRE [7].

Our hypothesis is that the autoreactive T cell repertoire may be important for tumour immunosurveillance. This was tested by challenging AIRE deficient mice, that have an enlarged and diverse autoreactive repertoire, with melanoma, and subsequently characterising their immune responses.

Here, we characterised responses against the self-antigens of this natural anti-tumour repertoire, and investigated their thymic selection. Understanding the mechanisms that allow effective tumour immune responses using the naturally occurring autoreactive repertoire will be important in developing successful tumour immunotherapies against melanoma.

## Results

### APECED patients harbour anti-tumour antibodies

**Figure 1:**
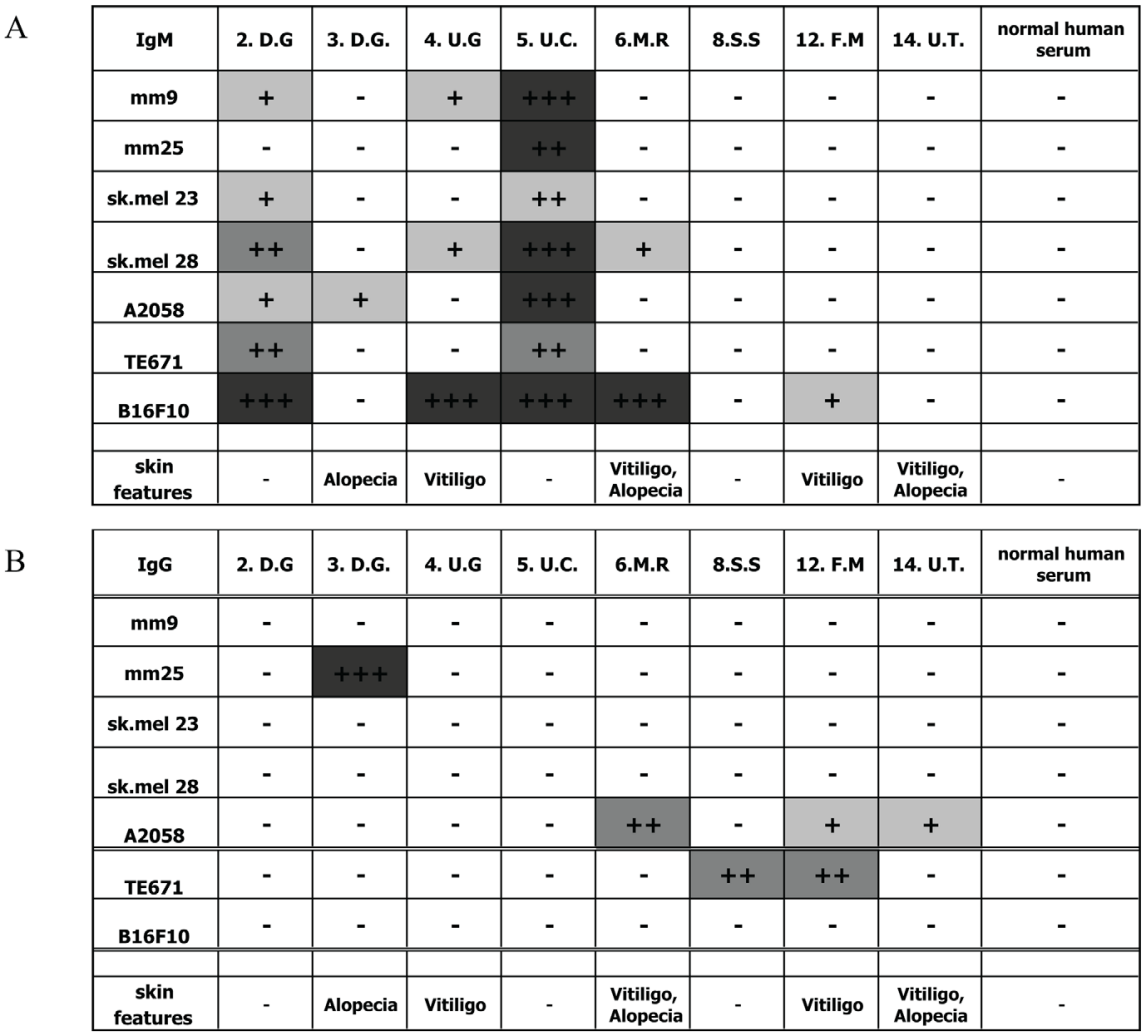
APECED patients with skin features have increased anti-melanoma antibody levels. Cell lines were stained with serum obtained from APECED patients (2.D.G, 3.D.G., 4.U.G., 5.U.C., 6.M.R., 8.S.S., 12.F.M., 14.U.T.) diluted 1/500. Mm9, mm25, sk.mel23, sk.mel28, A2058 are human melanoma lines, TE671 a human rhabdomyosarcoma, and B16F10 murine melanoma cell line. Background using normal human serum was substracted and mean fluorescence indicated as follows −<0.5, +0.5–0.75, ++0.75–1, +++>1. The secondary antibodies used were **A** anti-human IgM FITC and **B** anti-human IgG FITC.

The spontaneous immune reactivity towards tumours was tested with sera obtained from 8 APECED patients, which have a broader autoreactive repertoire than normal. As some of these patients had known skin reactivity, we tested for the presence of IgM and IgG antibodies against 5 different human melanoma cell lines and other transformed control cell lines (murine or human) by flow cytometry, and subtracted the background levels obtained with normal human serum. 5 out of 8 patients had detectable specific IgM antibodies to at least one human melanoma line, mostly several, irrespective of whether their symptoms included skin features or not (Fig. 1A). APECED patients sera also stained the human rhabdomyosarcoma cell line TE671 and the murine melanoma B16F10. Such broad reactivity is often seen with low affinity antibodies, especially IgMs and is not surprising in APECED patients where a wide range of auto-antibodies has been described.

When we tested for melanoma-specific IgGs, these were found in only 5 out of 8 patients (usually without concomitant IgMs) (Fig. 1B). Interestingly, 4 of these patients displayed clinical symptoms involving the skin and/or hair follicles, suggesting that their vitiligo or alopecia may be related to melanocyte destruction. Two patients had elevated IgG levels to the human rhabdomyosarcoma TE671 cells, but none showed reactivity to the murine B16F10 melanoma cell line (Fig. 1B).

### AIRE deficient mice reject tumours after priming

To test the hypothesis that an expanded autoimmune repertoire is important in mediating tumour immunosurveillance, we challenged AIRE^−/−^ mice (backcrossed onto C57Bl/6 mice for 10 generations) with the poorly immunogenic, syngeneic melanoma line B16F10. Importantly and as described elsewhere, the autoimmune phenotype is less pronounced in the C57Bl/6 strain as compared to other strains [8]. Yet, in a proportion of mice, we found immune infiltrates in several organs, such as stomach and salivary glands (data not shown). Moreover, ageing AIRE^−/−^ females were sterile.

**Figure 2:**
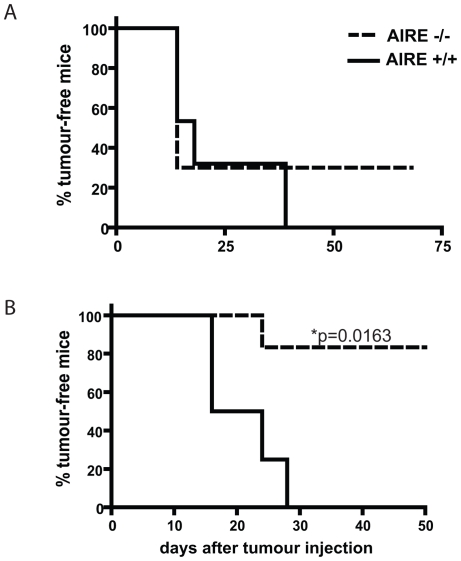
AIRE^−/−^ mice reject tumour more efficiently than AIRE^+/+^ littermate controls mice after priming. **A** Mice of indicated genotype were challenged with B16F10, and tumour growth monitored over 75 days. **B** As treated in **A**, but mice were primed with irradiated B16F10 4 weeks prior to challenge. Curves were compared with Logrank test with p values indicated. Similar trends were found in at least 5 different experiments. In each of these experiments between 5 and 10 mice/group were included.

Young adult AIRE^−/−^ or littermate controls AIRE^+/+^ or AIRE^+/−^ mice were challenged with 2×10^5^ B16F10 s.c. Usually, in non-primed naïve animals, there was no overall significant difference in tumour growth between the different genotypes. However, in some experiments, up to 30% of AIRE^−/−^ mice remained tumour-free while all AIRE^+/+^ mice developed tumours. (Fig. 2A). In contrast, when mice were primed with 10^7^ lethally irradiated B16F10 tumour cells 4 weeks prior to challenge with live tumour cells, up to 80% of AIRE^−/−^ mice remained tumour–free as opposed to only 20% of AIRE^+/+^ animals (Fig. 2B). Since we observed no difference between wild type and heterozygote (het) mice, the latter were not always included in subsequent experiments. We occasionally detected a depigmentation of the fur at the site of tumour cell injection (in approximately 20% of all mice). In addition, development of a lighter coat colour starting from the neck and spreading over the whole body could be detected in AIRE^−/−^ mice only (in approximately 20% of these).

To test whether this was a melanoma-specific effect, we also challenged the AIRE^−/−^ mice with the Lewis lung carcinoma 3LL after priming with 3LL four weeks earlier. No difference in tuour growth rate was detected between AIRE^−/−^ and AIRE^+/+^ mice, probably due to the fast growing nature of the 3LL tumour.

### AIRE^−/−^ mice produced increased titers of non-protective anti-tumour antibodies

**Figure 3:**
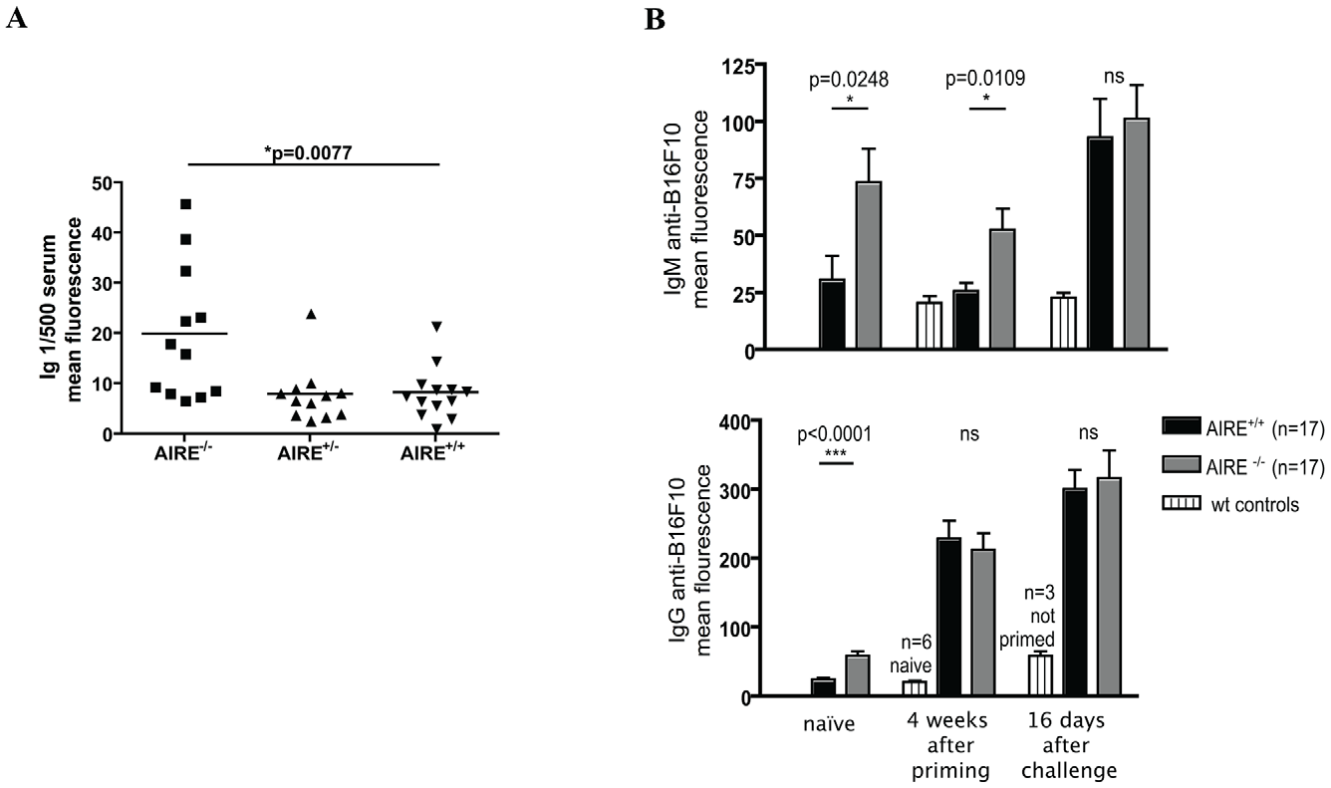
Anti-B16F10 antibodies serum levels are elevated. Mice of the indicated genotype were primed with 5×10^6^ irradiated B16F10 and challenged with 2.5×10^5^ live B16F10. **A** At time of sacrifice (when tumours reached 10 mm^2^ or in the case of tumour-free mice after 50 days), serum was obtained. 1/500 diluted serum was used to stain B16F10. Secondary antibody was an anti-mouse pan Ig APC. **B** Serum was obtained before priming, 4 weeks after priming, or 16 days after live tumour challenge and used to stain B16F10. Naïve littermate controls or unprimed but challenged controls were included were indicated. Secondary antibodies used were anti-mouse IgM FITC (upper panel) or anti-mouse IgG FITC (lower panel). * p value by Mann-Whitney test comparing mean fluorescence of serum staining B16F10. These findings were reproduced three times with similar results with n>10 in each group.

The detection of antibodies against melanoma in APECED patients, and the fact that primed AIRE^−/−^ mice rejected B16F10, prompted us to look for B16F10-specific antibodies in the serum of AIRE^−/−^mice. Serum was obtained at time of sacrifice from naïve mice, from mice primed with irradiated B16F10 cells (4 weeks post-priming), and once more from primed mice 16 days after challenge with alive B16F10 tumour cells. B16F10 cells were stained with the murine sera and analysed by flow cytometry. Overall B16F10-specific immunoglobulin (Ig) levels were significantly increased in the serum of AIRE^−/−^ mice compared to AIRE^+/−^ or AIRE^+/+^ littermate controls (Fig. 3A). When specifically stained for IgM and IgG, IgM antibodies were found significantly increased in AIRE^−/−^ mice even before priming with tumour cells, a trend that was maintained after tumour challenge. As expected, IgG responses increased significantly after tumour challenge (Fig. 3B). No significant changes were observed after tumour challenge between AIRE^−/−^ and AIRE^+/+^ mice. Polyclonal anti-melanoma titers of the isotype-switched mature IgGs are increased by antigen exposure in both types of mice, indicating that the relevant T helper cells are functional in the absence and presence of AIRE. However, IgM and IgG titers are significantly increased before priming in AIRE^−/−^ mice, as compared to AIRE^+/+^ mice suggesting that T helper cells have been exposed to melanoma antigens, presumably expressed on melanocytes, before antigenic challenge with melanoma. This hypothesis is confirmed by the APECED serum data. We conclude that the rejection of melanoma in primed animals is not mediated by IgG antibodies, which was also confirmed by the inability of AIRE^−/−^ serum to confer significant protection against a B16F10 challenge in wild type hosts (data not shown). Similar IgM and IgG titers (at all three time points) were found using the 3LL model (data not shown), in which the AIRE^−/−^ mice did not show tumour protection, corroborating that antibodies probably do not mediate tumour rejection.

### AIRE^−/−^ display a higher proportion of activated T cells in lymphoid organs

**Figure 4:**
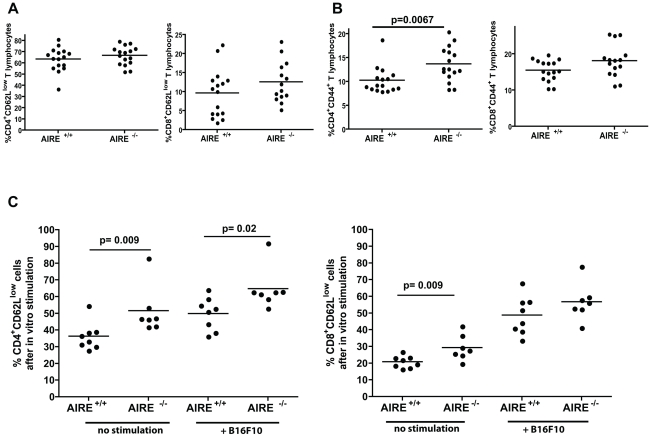
AIRE^−/−^ mice have increased overall CD4 and CD8 responses after tumour challenge. Mice of indicated genotype were primed and challenged with B16F10, then splenocytes were isolated, and stained for CD62L or CD44. **A** Percentage of CD62L^low^ cells among CD4^+^ or CD8^+^ T lymphocytes ex vivo **B** Percentage of CD44^+^ cells among CD4^+^ or CD8^+^ T lymphocytes ex vivo **C** Percentage of CD62L^low^ cells among CD4^+^ or CD8^+^ T lymphocytes after in vitro culture with IFNγ stimulated B16F10. * p value Mann-Whitney test comparing % of cells with low CD62L among CD4^+^ CD3^+^ T lymphocytes. This result is representative of two repeats.

To test the involvement of T cells in the observed anti-tumour response in AIRE^−/−^ mice, we measured frequencies of activated CD4^+^ and CD8^+^ T cells following tumour challenge in lymph nodes (LN). We first determined the percentage of activated T cells after tumour challenge, by measuring the frequencies of CD44^+^ and CD62L^low^ cells. While *ex vivo* neither CD4^+^ nor CD8^+^ cells showed a significant downregulation of CD62L (Fig. 4A), CD44 was significantly upregulated on CD4^+^ T lymphocytes and in a fraction of mice on CD8^+^ cells (Fig. 4B). We then stimulated splenocytes from tumour-challenged mice for 48 h with IFNγ-treated B16F10 and then stained with anti-CD62L antibodies. Treatment of B16F10 with IFNγ enhanced MHC class I and II expression levels significantly (data not shown), and therefore likely also antigen presentation. Interestingly, after the *in vitro* culture period, we observed significantly more activated CD8^+^ and CD4^+^ T cells among the splenocytes obtained from AIRE^−/−^ mice compared to AIRE^+/+^ mice as judged by their loss of CD62L expression (Fig. 4C, no stimulation). After restimulation, AIRE^−/−^ splenocytes showed significantly more CD62L^low^ cells than AIRE^+/+^ splenocytes (Fig. 4C+ B16F10) suggesting that CD4^+^ and to a lesser degree CD8^+^ T lymphocytes are activated after tumour challenge in AIRE^−/−^ mice presumably because of less efficient thymic clonal deletion in the absence of AIRE.

To determine whether tumour protection is mediated by CD4^+^ or CD8^+^ T cells, we transferred 10^7^ CD4^+^ T cells or 1.5×10^6^ CD8^+^ T cells from B16F10 challenged AIRE KO or AIRE WT mice into naïve hosts, followed by challenge with B16F10 the following day. Neither transfer offered a significant delay in tumour growth, although CD4^+^ T cells did delay the tumour onset by a few days, but then all mice succumbed to tumours. It is possible that a combination of different T cell subsets in combination with serum may confer protection.

**Figure 5:**
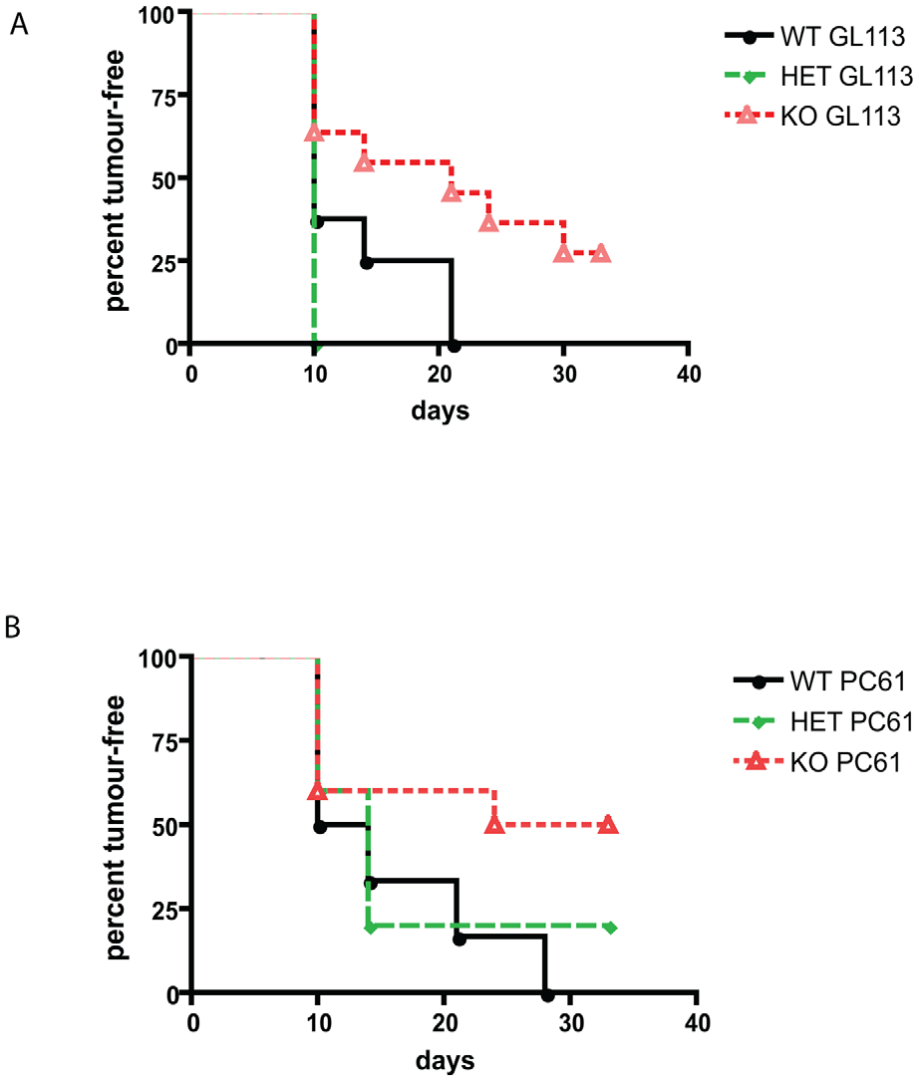
Depletion of regulatory T cells leads to improved tumour rejection in both AIRE^−/−^ and AIRE^+/+^ mice. Mice of the indicated genotype were primed and challenged with B16F10. Three days and one day prior to challenge, mice were treated twice with 0.5 mg of control antibody GL113 (**A**), or with regulatory T cell-depleting antibody PC61 (**B**). Seven days after depletion with PC61 but not GL113 antibodies, CD4^+^CD25^+^ cells were reduced by 60% in the blood. Appearance of tumours was measured for 121 days.

It has been suggested that the generation of regulatory T cells (Tregs) may be dependent upon AIRE and that the absence of AIRE may result in the generation of fewer or less functional Tregs [7]. A decrease in functional Treg cells would lead to higher T cell responses, thereby possibly explaining the improved tumour rejection seen in AIRE^−/−^ mice. To investigate this, the frequencies of Tregs were determined in AIRE^−/−^ and littermate control mice before and after tumour challenge. However, no differences in the numbers of CD25^+^ CD4^+^ FoxP3 cells in draining LN (dLN), TILs and spleen were found between the different groups (data not shown). The functional capacity of AIRE^−/−^ Treg cells was assessed by depleting them prior to tumour challenge with an anti-CD25 monoclonal Ab (mAb). This led to a slight improvement in tumour rejection by littermate controls [9], as expected, but also similarly enhanced tumour rejection by AIRE^−/−^ mice (Figure 5). These results suggest that the numbers and function of Tregs, inhibiting anti-tumour responses, are normal in AIRE^−/−^ mice. Evidently, the tumour rejection seen in AIRE^−/−^ mice is not due to a lack of regulatory T cells or their activity alone.

### AIRE^−/−^ mice mount anti-tumour T cell responses only against specific tumour self-antigens

**Figure 6:**
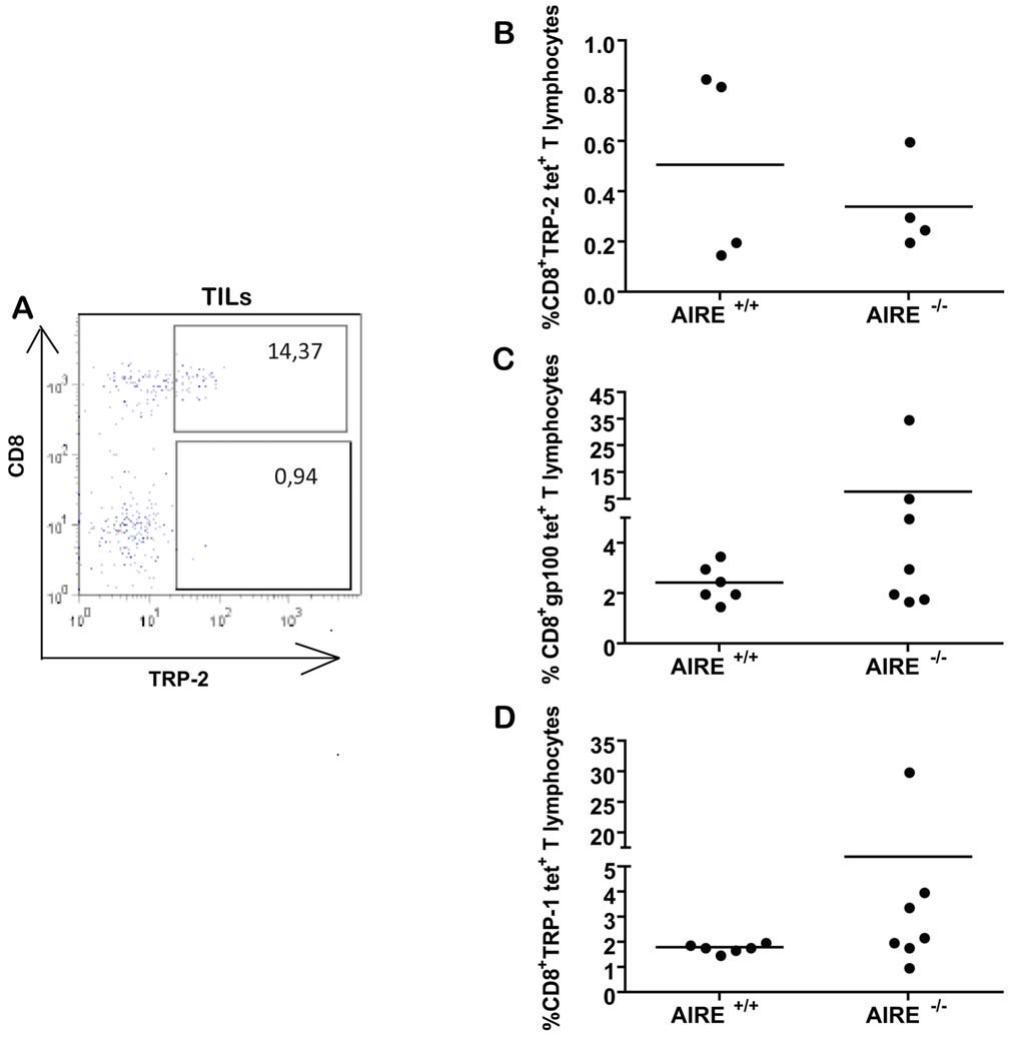
MAA-specific CD8^+^ T cells present in TILs and draining LNs. **A** Wildtype mice were injected with 10^6^ live B16F10 and 10 days later, TILs were isolated and stained for CD8 and TRP-2-specific TCR using tetramers. **B** and **C** Mice of indicated genotypes were primed and challenged with B16F10 cells, then frequencies of gp100, TRP-1 or TRP-2 tetramer positive cells in the CD8^+^ T cell population were plotted. Representative of two similarly conducted experiments.

To further characterize the anti-tumour response of AIRE^−/−^ mice, we analysed the antigen specificity of the T cell populations found among tumour-infiltrating lymphocytes (TILs) (when tumours grew) and in dLN. As CD8^+^ T cells were activated in response to tumours (Fig. 4) and MHC class I tetramers are more readily available, we concentrated on CD8^+^ T cell responses. Antigen-specific CD8^+^ T cells were identified by MHC class I tetramers for MAAs (mouse gp100, TRP-1, TRP-2). Surprisingly, amongst the TILs of control mice, up to 14% of all CD8^+^ T cells were found to be specific for a single epitope derived from TRP-2 (representative staining shown in Fig. 6A). In the tumour-dLN, gp100, TRP-1 and TRP-2 -specific CD8^+^ T cells were also detectable, whereas none were found in the spleen. As the majority of AIRE^−/−^ mice remained tumour-free, we measured gp100, TRP-1 and TRP-2- specific CD8^+^ T cells in dLN. In approximately half the mice, frequencies of either gp100- or TRP-1-specific CD8^+^ cells were increased in AIRE^−/−^ mice compared to those in littermate control mice (Fig. 6C and B). Higher responses in AIRE^−/−^ mice were observed consistently in several experiments however, due to the wide variations seen among AIRE^−/−^ mice, the difference did not reach statistical significance.

**Figure 7:**
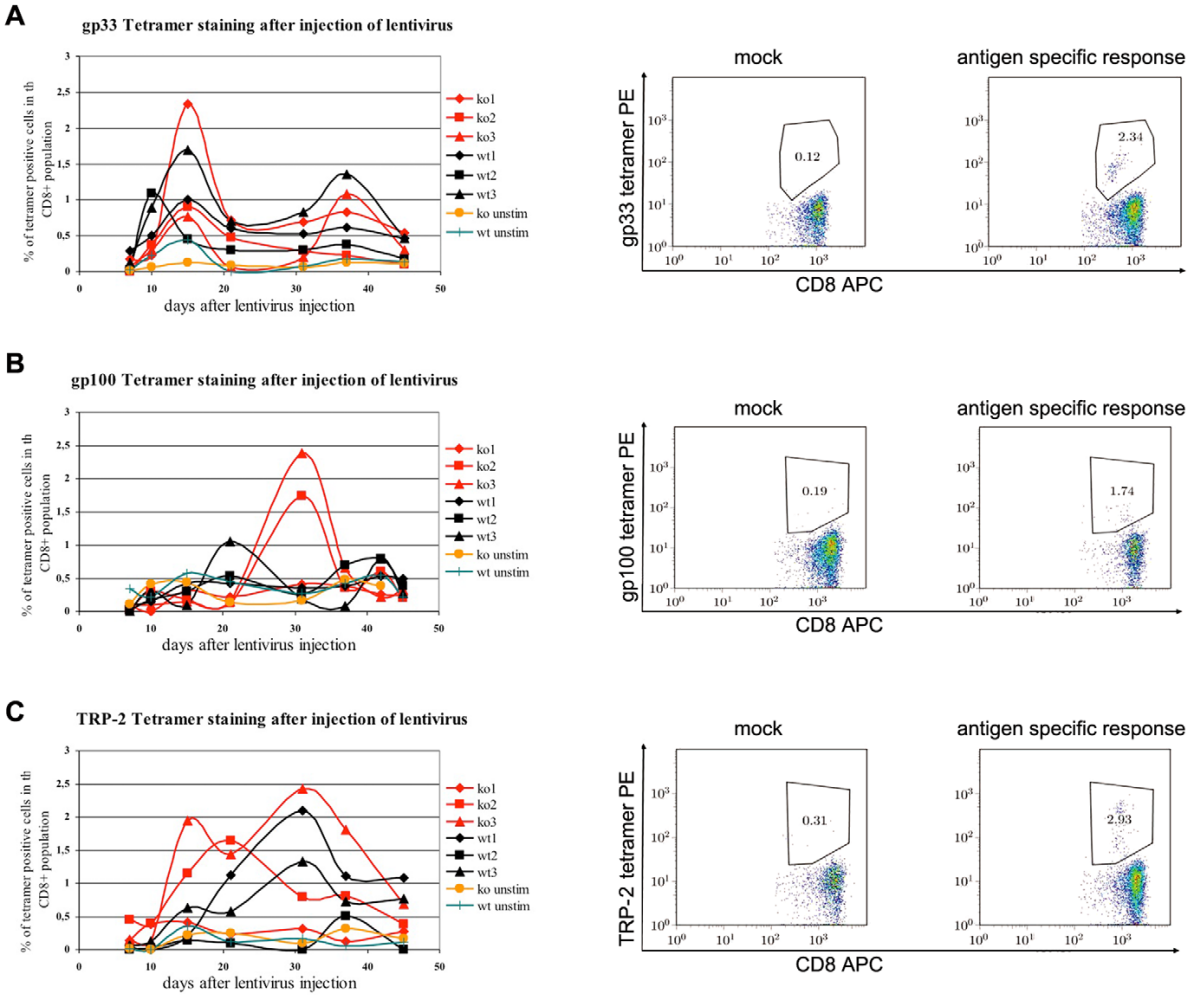
AIRE^−/−^ mice do not hyperreact to foreign antigen but respond better to certain melanocyte antigen than AIRE^+/+^ littermate controls. Mice of the indicated genotype were injected s.c at the base of the tail with 4×10^6^ pfu of lentivirus expressing the indicated peptides. Serial samples of blood cells (left panels) were stained for CD8 and MHC class I tetramers specific for T cells responding to the injected peptides (example at peak of response in right panels). **A** LCMV-derived foreign gp33 presented by H-2D^b^ **B** mouse TRP-2 presented by H-2K^b^ **C** gp100 presented by H-2D^b^.

Notably, we observed a similar frequency of TRP-2-specific CD8^+^ T cell responses in AIRE^−/−^ and AIRE^+/+^ mice (Fig. 6B). To elucidate this discrepancy we measured the responses to each of these antigens in a more controlled system using lentivirus expressing either the gp100, TRP-2 or gp33 epitopes to stimulate antigen-specific responses *in vivo*. The model foreign antigen gp33, an epitope derived from the LCMV glycoprotein, was included to address whether tumour rejection by AIRE^−/−^ mice could be due to a generally hyperactive immune system in these. Mice were challenged once at the base of the tail with the lentivirus and numbers of specific CD8^+^ T cell in the blood were determined by tetramer staining over 45 days. Examples of these stainings are shown in the right panels of Figure 7. When challenged with a foreign antigen, lentiviral priming induced a biphasic immune response usually peaking after two weeks and 5 weeks [10].

Not surprisingly, naïve unstimulated control, AIRE ko or wt mice showed no significant responses to any of the antigens tested (Fig. 7A–C). As expected AIRE^−/−^ and littermate controls displayed similar CD8^+^ T cell numbers in response to lentivirus expressing the foreign antigen gp33 and in a biphasic pattern peaking first at around day 15 and then 38. This suggests that AIRE^−/−^ mice are not hyper-reactive, at least not to this particular foreign antigen with their CD8^+^ T cell response (Fig. 7A).

A similar biphasic response was observed for the MAA self-antigen TRP-2 and as expected, 2/3 of both AIRE ko and control mice mounted a TRP-2-specific CD8 response (Fig. 7C) [10]. Following immunization with the lentivirus expressing gp100, 2/3 AIRE ko mice mounted a good response. In contrast only one wt mouse made even a weak response (Fig. 7B). Together these results suggest that the expansion of TRP-2-specific T cells is AIRE-independent and occurs equally in the periphery of AIRE^+/+^ and AIRE^−/−^ animals. By contrast, gp100-specific T cells were rare in littermate control animals, but were readily induced following lentiviral immunization of AIRE ko mice, suggesting a greater expansion of gp100-specific T cells in the absence of AIRE.

### T cell responses to tumour self-antigens are dependent on thymic expression of those antigens

As T cell responses to gp100 but not TRP-2 were generated more efficiently in the absence of AIRE, we investigated the expression of gp100 and TRP-2 mRNA in the thymic epithelium of young AIRE^+/+^ and AIRE^−/−^ mice. mRNA was extracted from flow sorted mTECs (G8.8^high^Ly51^low^) and cTECs (G8.8^high^Ly51^high^) from 3 AIRE^+/+^ and 3 AIRE^−/−^ mice and gp100 and TRP-2 mRNA amplified. mRNA from melanoma B16F10 cells expressing the MAAs gp100 and TRP-2 cells was included as a positive control and GAPDH was used for normalisation.

**Figure 8:**
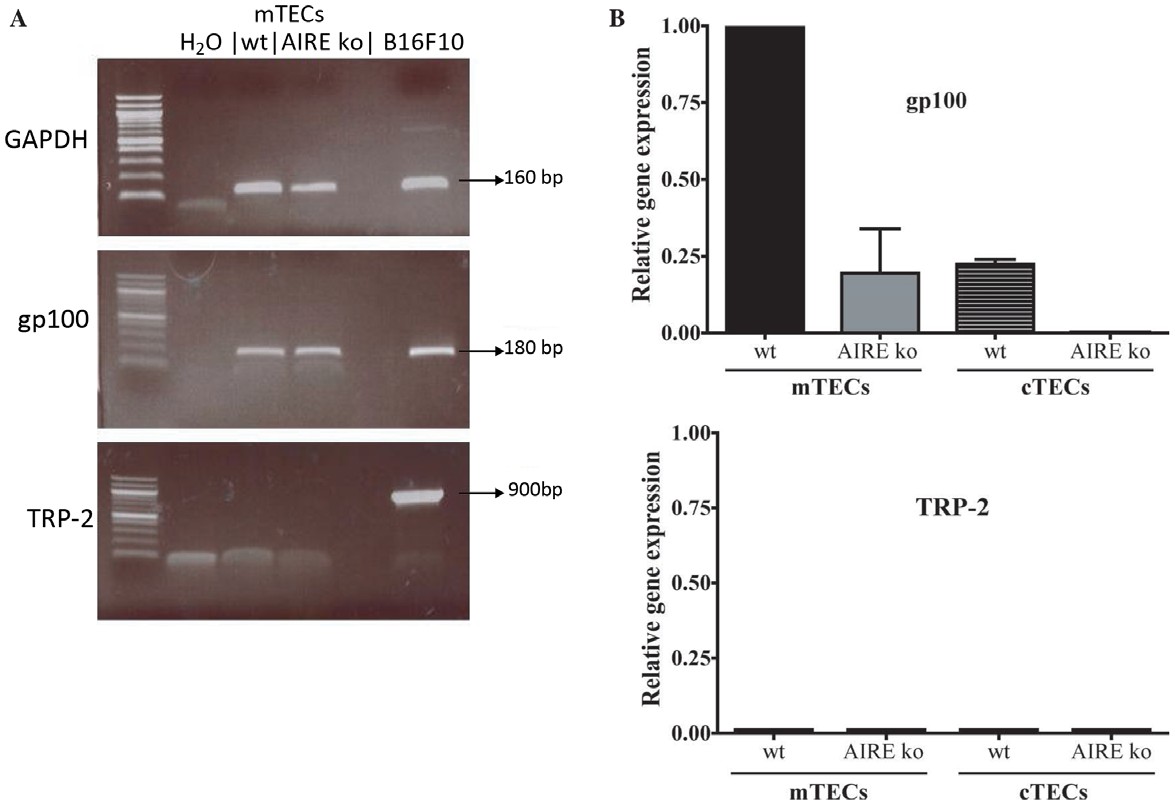
Expression of melanocyte antigens in medullary thymic epithelial cells. Medullary or cortical thymic epithelial cells were sorted by flow cytometry. **A** RT-PCR was performed on both types of cells using primers for GAPDH, gp100 and TRP-2. **B** Real time qPCR was performed using sorted populations. Expression relative to gp100 expression in AIRE^+/+^ mTECs is shown for gp100 and TRP-2. Error bars are from duplicates.

By non-quantitative RT-PCR, the RNA extracted from B16F10 yielded bands for TRP-2 and gp100 at the expected sizes (Fig. 8A). Interestingly TRP-2 mRNA was not at all detected in mTECs obtained from AIRE^+/+^ or AIRE^−/−^ mice. However, in this non-quantitative PCR, gp100 was found equally expressed in both littermate controls and AIRE^−/−^ mice (Fig. 8A). To obtain a better and quantitative resolution of mRNA levels, we then used real time qPCR to quantify the MAA expression in the two TEC subpopulations. The levels of gp100 were highest in mTECs from littermate controls, and were ∼4 fold lower in AIRE^−/−^ mTECS. As reported previously gp100 levels were lower in AIRE^+/+^ cTECs [11], and undetectable in AIRE^−/−^ cTECs (Fig. 8B upper graph). Confirming the RT-PCR, TRP-2 was undetectable in all the TEC samples tested using qPCR (Fig. 8B lower graph). These findings may explain why we detected responses to TRP-2 after both lentiviral and tumour challenges irrespective of AIRE expression, and why gp100 responses were only detectable in AIRE^−/−^ mice.

## Discussion

Using AIRE ko mice that have an expanded autoreactive repertoire, we show that self-antigens alone can protect against a syngeneic melanoma challenge. Although this has been addressed before using MAA specific TCR transgenic models [12] [13] [14], this is the first time that the natural autoreactive TCR repertoire, unleashed by deficient thymic selection, has been demonstrated to be effective in melanoma rejection.

### Tumour resistance may depend on autoimmune repertoire

Even though the autoimmunity in AIRE^−/−^ mice is milder on the C57BL/6 background than in other strains [8], this autoreactive repertoire is sufficient to mediate tumour rejection. Our own data and other studies show that only a proportion of AIRE^−/−^ mice on the C57BL/6 background develop detectable autoimmunity to organs such as retina, stomach and salivary gland [8], [15]. Involvement of the skin has not been specifically examined in mice, but vitiligo and alopecia are prominent features in many APECED patients (Fig. 1). The heterogeneity of the autoimmune manifestations clearly also applies to the immune response and resistance to melanoma cells, seen in only ∼half of the mice. Interestingly, discolouring of the fur and white fur at the injection site was usually only observed in mice that rejected tumours confirming that when (mild) autoimmune signs occurred it correlated with effective immune responses. The variation in the autoimmune symptoms and anti-tumour responses may be due to the stochastic nature of both AIRE activity and T cell receptor rearrangement. For example in the non-responding mice, the relevant tumour antigen-specific TCR may not have been generated. These stochastic events as well as modifier genes [15] have been used to explain the notorious heterogeneity in organs affected even within APECED siblings [7].

From our data we conclude that both CD4 and CD8 T cells of the immune system are contributing to tumour rejection by the autoreactive immune repertoire. Anti-tumour antibodies are increased in AIRE^−/−^ mice, but they are not CD4 dependent (IgM) and are not protective. In contrast, auto-antibodies and auto-reactive B cells detected in the absence of AIRE expression appear to be crucial in the murine autoimmune pathology [16]. Notably, auto-antibodies against the transcription factors SOX9 and SOX10, important in the development of melanocytes, have been detected in APECED patients and are highly associated with skin symptoms such as vitiligo [17]. Similarly, auto-antibodies from APECED patients also stain the cytoplasm of keratinocytes and melanocyte nuclei, correlating with alopecia and vitiligo in these patients [18]. The localization of antigens identified so far is intracellular, whereas here we describe antibodies to melanoma surface molecules. Whether these antibodies target antigens on non-transformed melanocytes remains to be confirmed, but if they did, it would help to explain destruction of these melanocytes by antibodies, which is more difficult in the case of intracellular antibody targets.

It has been suggested that AIRE controls the positive selection of Tregs [7]. In the absence of AIRE, a lack of functional regulatory T cells may therefore explain the improved tumour rejection [19]. However, when Tregs were removed from AIRE^−/−^ mice, we found a further increase in tumour rejection. Failure to detect a difference in numbers of regulatory T cells is reminiscent of other studies using AIRE^−/−^ mice. Taken together we cannot exclude that AIRE may influence regulatory T cell development, however, the tumour rejection by AIRE^−/−^ mice cannot be solely explained by the lack of functional regulatory T cells.

We usually found a tumour incidence of ∼70% in non-primed AIRE^−/−^ animals and of only ∼20% in primed AIRE^−/−^ animals (Fig. 2). The fact that AIRE^−/−^ mice show increased IgG titers even before priming suggests that initial priming may have occurred in the absence of tumours on melanocytes, initiating some immune response. However, priming with irradiated melanoma cells may be necessary to provide efficient costimulation through cross-presentation of tumour antigens taken up from dying cells to initiate a mature and specific response. Such priming may be required to break the peripheral tolerance of the autoimmune repertoire. We found previously that a period of 4 weeks between priming and melanoma injection is ideal for the immune system to expand in time to reject challenge doses of live tumour cells, which otherwise grow into sizeable tumours within days [20].

A recent report attributed increased responses of AIRE ko mice to the model antigen hen egg lysozyme to a hyperreactive immune system [21]. However, the very similar responses we measured here in AIRE ko and wt mice to a lentivirus–expressed foreign LCMV epitope strongly argues against any general anti-tumour CD8^+^ T cell-mediated hypereactivity.

### Extending our conclusions to other tumours

As B16F10 is a very aggressive tumour, we suspect that the autoreactive repertoire might be sufficient to fully reject less aggressive and more immunogenic tumours. We therefore injected the fibrosarcoma-inducing drug methylcholanthrene (MCA) into 12 AIRE ko and 12 wt mice (data not shown). We only detected a small difference in tumour incidence between the two groups (42% in AIRE ko versus 55% of wt mice). However, as recommended by Schreiber et al [22], we focused on the kinetics of tumour growth. The first tumours were detected in ko mice at day 159 as opposed to day 145 in wt mice, i.e a very modest two week delay. The day at which half of the mice developed tumours (median) was 187 days in wt mice versus 268 days in ko mice. This ∼5 months latency may partly reflect innate NK mediated tumour control. That full rejection of MCA-induced tumours was not achieved may be due to the need of priming, though, unfortunately that would be precluded in this spontaneous model due to individual variations in MCA induced tumour antigens.

In addition, when the tumours had grown, the mice were approaching 12 months in age, by when their responsiveness may be declining, as we have noted to the melanoma B16F10 in AIRE^−/−^ mice. Interestingly, very old AIRE^−/−^ mice have an increased risk of marginal-zone B cell lymphoma and other hematopoietic irregularities [23]. The uncontrolled and constant activation of the immune system may eventually lead to transformation of hematopoietic, in particular lymphoid cells. We cannot explain why the autoreactive repertoire is unable to control the development of tumours in older mice. Oral carcinomas are well known in APECED patients [24]. However, their associated chronic candidiasis may be a major confounding local risk factor as no increased incidence of other carcinomas has been reported.

We found no protection in AIRE^−/−^ mice when we tested this model with the Lewis lung carcinoma 3LL. It is possible that the enlarged autoreactive repertoire may confer resistance to melanoma and less so to other tumours. The following observations are consistent with this hypothesis: Firstly, in healthy humans, there is a large pool of peripheral CD8^+^ T cells of naïve phenotype specific for Melan-A [25]. Secondly, vitiligo is an autoimmune skin-depigmenting disorder characterised by loss of epidermal melanocytes. It is frequently observed in melanoma patients [26] and is associated with a good prognosis [27]. Thirdly certain AIRE single nucleotide polymorphisms associated with reduced AIRE stability are significantly more frequent in healthy patients than in melanoma patients [26] and so are Melan A-specific T cells, again implying protective effects of AIRE deficiency.

### Implications for selection of tumour vaccine targets

We show in this study that gp100-specific T cells were readily induced in AIRE^−/−^ but not in littermate controls, whereas T cells specific for TRP-2 were induced in both. This difference correlated with the clear AIRE-dependent expression of gp100 in TEC [11], and concomitant absence of TRP-2; also with more robust CD8^+^ responsiveness of AIRE^−/−^ mice to both gp100 lentivirus (Fig. 7) and tumour challenge. Surprisingly, TRP-2 was undetectable in both wt and AIRE^−/−^ mTECs; however, that explains the equally strong responses to lentivirus-encoded antigen in both AIRE^+/+^ and AIRE^−/−^ mice. As T cells to TRP-2 were not sufficient to reject the tumour, they are presumably regulated by peripheral tolerance mechanisms. Indeed, firstly transferred TRP-2-specific transgenic T cells were not able to reduce subcutaneous tumour burden [14] unlike TRP-1 and gp100-specific TCR transgenic T cells [12], [13]. Secondly TRP-2 specific T cells although easily elicited by lentivirally expressed TRP2 express more PD1 and fail to produce cytokines [10]. In summary we hypothesize that as TRP-2 may not have induced clonal deletion in the thymus, peripheral tolerance mechanisms are likely to be in place.

A recent study in human thymus showed that TRP-2 was expressed in almost all samples tested, albeit with a large variation in levels of expression, whereas gp100 mRNA was not always detected [28]. These differences between human and murine expression may just be explained by the stochastic nature of AIRE activity, and by the differences in the melanin biosynthesis pathway and melanocyte numbers in human versus murine skin (mice only have very few melanocytes in their skin).

There are several reports showing AIRE expression in peripheral cells including DCs and stromal lymph node cells, although the functional consequences of peripheral AIRE expression is poorly understood [29]. One such example of a role of AIRE outside the thymus is the finding by Niki et al that AIRE^−/−^ NOD mice do not destroy their beta cell islets and do not develop diabetes as predicted but rather produce auto-antibodies against a protein expressed by acinar cells, another type of pancreatic cells, and this protein is not under the transcriptional control of Aire in the thymus [30]. This suggests that Aire may regulate the survival of autoreactive T cells beyond transcriptional control of thymic expression, a mechanism that could well operate here. To add to this complexity, other transcription factors have been shown to exert a similar role to AIRE in the periphery such as Deaf [6]. To shed light on this in respect to tumour antigens demands further investigation.

To our knowledge, this is the first investigation to highlight the need to know the candidate antigens and their dependence on AIRE for planning anti-melanoma vaccine strategies. We conclude that the immune system establishes tolerance to tumour antigens in at least three ways: (**i**) To some antigens not expressed in the thymus, tolerance is maintained solely in the periphery, like murine TRP-2. (**ii**) Others like gp100 and several thousand others [7] are expressed in the thymus under qualitative or quantitative influences of AIRE. Specific loss of AIRE-dependent thymic expression of a single antigen leads to the development of autoimmunity in the organ where this antigen is expressed [31] [32]. (**iii**) Yet other antigens may be expressed in the thymus thanks to other regulators analogous to AIRE. The existence of the latter type has been confirmed for non-MAA antigens such as C-reactive protein and GD67 [29] and on a broader level in a study by Derbinsky et al [33]. However, this categorization demands further investigation as does the precise role of AIRE in establishing tolerance. Clearly it is important to know the category (i to iii) to which the candidate tumour antigen belongs as well as the peripheral tolerance mechanisms to which it is subject.

It is well known that occasional autoreactive T cells escape thymic deletion even when AIRE is expressed normally [25]. Why does the thymus not eliminate these autoreactive T cells to avoid autoimmunity? The conventional explanation for this leaky thymic selection is that the broader the T cell repertoire, the more likely it is to include TCRs cross-reactive with a large variety of foreign antigens encountered later in life. Fortuitously (or purposefully), a few of the escapees may help to fight tumours, but some autoimmune collateral damage (e.g. to normal melanocytes) may be inevitable. Indeed, there are several examples in humans of effective spontaneous protective anti-tumour responses accompanied by autoimmune symptoms. For example, autoimmune paraneoplastic neurological disorders (PND) result from anti-tumour responses against neuronal antigens that are aberrantly expressed in cancer cells. Although they may cause autoimmune neurological damage, the tumour prognosis is often strikingly better than in tumour patients with no PND [34]. Clearly, T and B cell responses directed at tumour autoantigens are capable of rejecting tumours. Whether they were originally selected purposefully to reject tumours needs further confirmation.

## Materials and Methods

### Ethics statement

Human serum were diagnostic samples obtained with informed written consent and approved by the local ethics committee USL 8, Cagliari, and Hospital Healthcare Management of Microcitemico Hospital, Clinic Pediatric II, Cagliari for the research project entitled “APECED, a common disease in the Sardinian population 1) genotype/phenotype correlation. 2) Early diagnosis”, which includes this study's research aim.

All animal experiments were approved by Oxford University institutional review committee and were performed under the home office project licence PPL 30/2407. Animal suffering was kept to the minimum according to the Home office guidelines.

### Patients

The Sardinian APECED patients were all *AIRE* R139X homozygotes. Their clinical details are summarized in [35].

### Cells

Cell lines (TE671, B16F10, sk.mel23, sk.mel28, A2058) were purchased from ATCC. The human melanoma lines mm9 and mm25 were a kind gift from Vincenzo Cerundolo, Oxford University [36].

### Animals

Mice were bred and housed in Biomedical Services in Oxford (BMS). They were kept in individual ventilated cages. The AIRE ko mice and genotyping protocol were kindly provided by the late Leena Peltonen, Finland and were backcrossed to C57BL/6 mice for over 10 generations. All mice used for this study, AIRE^−/−^, AIRE^+/−^ and AIRE^+/+^ had undergone the same number of backcrossings and age and sex-matched littermates were used in all experiments. All animal experiments were approved by Oxford University institutional review committee and were performed under the home office project licence PPL 30/2407. Animal suffering was kept to the minimum according to the Home office guidelines.

### Mouse genotyping

The insertion of the neo-cassette into the *AIRE* gene was tested with the following primers:

**3.1F** 5′ TGA GAC AGT TCC TCT GTG TAG CTT TGG CTG TGG 3′

**4.1R** 5′ GGC GCT ACC GGT GGA TGT GAA ATG TGT 3′

**NEO5.2** 5′ TCT TGG GAC TTA CCT GGT TTA ACC TGG GGC TCA CTG 3′

Using all three primers and the GO Taq Green Master Mix (Promega, Madison, WI, USA), the PCR of genomic knock-out (ko) DNA yielded a 223 bp long fragment on both alleles whereas the wild type (wt) DNA amplified a 700 bp fragment, and in heterozygotes both fragments were amplified.

### Mouse challenge

B16F10 was grown in RPMI 1640, 10% fetal calf serum, L-glutamine and antibiotics. Adherent B16F10 were always lifted with PBS and centrifugation was avoided where possible. Mice were either challenged s.c. with 2×10^5^ B16F10 directly or first primed s.c. with 5 or 10×10^6^ irradiated B16F10 (12,000 rad) and then challenged with live B16F10 s.c (all injections in the flank) after 30 days. Mice were euthanized when their tumours reached 10 mm^3^ in size. When challenged with lentivirus, mice were injected at the base of the tail with 10^7^ pfu lentivirus containing either the irrelevant antigen of LCMV epitope mgp33_33–41_(KAVYNFATC) presented by H2-D^b^ or mTRP-2_180–188_ (SVDFFVWL) presented by H-2K^b^ or mgp100 (EGSRNQDWL) presented on H-2D^b^.

### Tumour infiltrating lymphocytes

When a maximum tumour size of 10 mm^3^ was reached, B16F10 melanomas were excised, tumour tissue cut into 1 mm^3^ pieces and cells isolated either with the gentleMACS dissociator (according to the manufacturer's instructions, Miltenyi Biotec, Bergisch Gladbach, Germany) or with collagenase and DNAse following a protocol from Quezada et al [37]. Cells were further gradient-purified on Lymphoprep (Axis-Shield, PoC AS,Oslo, Norway).

### Restimulation of primary cells

In addition to the *ex vivo* analysis, bulk splenocytes were restimulated with B16F10 (1∶1 ratio) that had upregulated both MHC class I and II molecules in response to 200 units IFNγ for 48 h. Cells were extensively washed prior to addition of splenocytes.

### Serum staining

Sera were used at 1/50, 1/500 and 1/5000 dilutions to stain the B16F10 cell line. FITC conjugated anti-mouse IgM or IgG (Invitrogen, Carlsberg, CA, USA) and FITC conjugated anti-human IgM (Sigma-Aldrich, Gillingham, UK) or IgG (Abcam, Cambridge, UK) were used to reveal specific staining.

### Antibody and tetramer staining

All primary antibodies were purchased from eBioscience (San Diego, CA, USA) and used at concentrations between 1 and 5 µg/ml, except for anti-CD62L (from Caltag lab, Burlingham, CA, USA). Secondary anti-human IgG FITC was purchased from Sigma-Aldrich (Gillingham, UK) and anti-human IgM FITC from Abcam (Cambridge, UK). Tetramer staining was performed with 2 µg/ml of gp33 (KAVYNFATA, D^b^), gp100 (EGSRNQDWL, K^b^) and TRP-2 (SVDFFVWL, K^b^) tetramers and with 2.15 µg/ml TRP-1 (TWHRYHLL, K^b^) tetramer at 37°C for 20 min and followed by surface marker staining.

### Medullary thymic epithelial cells

Thymi from 4–5 weeks old AIRE ko and wt mice were dissected and disaggregated using the gentleMACS dissociator (Miltenyi, Germany) using liver or lung protocols provided by manufacturer. The epithelial cell fraction was enriched by negative selection with the CD45^+^ leukocytes Isolation kit (Miltenyi, Germany). The unlabelled flow-through was collected and flow cytometry-sorted using rat anti-G.8.8 (Invitrogen, CA, USA), followed by goat anti-rat FITC, thoroughly washed, then stained with biotinylated rat anti-Ly51 and streptavidin PEcy7. mTECs were sorted according to G8.8^high^Ly51^low^ phenotype and G8.8^high^Ly51^high^ cells were sorted as cTECs [11].

### RT-PCR and qPCR

Messenger RNA was isolated from cTECs, mTEcs or B16F10 using the RNeasy Micro kit (Qiagen, Hilden, Germany), reverse transcribed using the Omniscript RT kit (Qiagen, Hilden, Germany) adding 5 µg (when possible) of denatured RNA to the kit components according to the manufacturer's instructions.

The following primers were included in the PCR reaction (annealing temperature 62°C).

GAPDH forward 5′ CCT GGA GAA ACC TGC CAA GTA T 3′

  reverse 5′ AGA GTG GGA GTT GCT GTT GAA G 3′

gp100 forward 5′ ACA TTT CAT CAC CAG CAG GGT GCC 3′

  reverse 5′ AAC AAG TG GGT GCT GGC C 3′

TRP-2 forward 5′ CCA TTG ATT TCT CTC ACC AAG GG 3′

  reverse 5′ TCT CTT GCT GCT GAG ACC TGT CTC 3′

For the qPCR, the Taq-Man gene expression system (Applied Biosystems, Foster City, CA, US) was performed using the pre-designed probes for TRP-2 (Mm00494496_m1), gp100 (Mm00498996_m1) and GAPDH (Mm99999915_g1). To normalise samples, the Ct value ( = the number of PCR cycle in which the product-specific fluorescence first exceeds background) for GAPDH was subtracted from Ct value for each gene. The relative gene expression was then calculated, setting one condition as standard according to the manufacturer's formula.
